# With or Without You?—A Critical Review on Pesticides in Food

**DOI:** 10.3390/foods14071128

**Published:** 2025-03-25

**Authors:** Tamara Lazarević-Pašti, Vedran Milanković, Tamara Tasić, Sandra Petrović, Andreja Leskovac

**Affiliations:** Department of Physical Chemistry, VINČA Institute of Nuclear Sciences—National Institute of the Republic of Serbia, University of Belgrade, 11000 Belgrade, Serbia; vedran.milankovic@vin.bg.ac.rs (V.M.); tamara.tasic@vin.bg.ac.rs (T.T.); sandra@vin.bg.ac.rs (S.P.); andreja@vin.bg.ac.rs (A.L.)

**Keywords:** sustainability, agriculture, health, environmental impact, integrated pest management

## Abstract

Pesticides are very important in modern agriculture, protecting crops against pests and diseases to ensure food safety. However, the use of pesticides in food production has raised significant concerns regarding their potential impacts on human health and the environment. This review provides comprehensive insights into the current status, future projections, and debates surrounding pesticides in food. Beginning with a historical overview of pesticide use in agriculture, the types of pesticides commonly used and the presence of their residues in food commodities are explored. The health and environmental impacts associated with pesticide exposure are examined, including both human health effects and ecological consequences. An analysis of the regulatory frameworks governing pesticide management at international and national levels is presented, along with emerging trends and future projections in pesticide technologies and agricultural practices. Strategies for mitigating pesticide risks, such as Integrated Pest Management and alternative approaches to conventional pesticide use, are discussed. Finally, the controversies surrounding pesticide use, including public perception, consumer concerns, and policy debates, are addressed. Through a critical examination of these issues, this review underscores a growing need for innovative solutions that can effectively balance agricultural demands with human health and the environment, enabling more resilient and sustainable food production.

## 1. Introduction

The significant rise in the global population during the 20th century was made feasible through a corresponding increase in food production. Pesticides play a vital role in modern food production systems, serving as essential tools to preserve crops from pests, diseases, and weeds. Their significance lies in ensuring global food safety and security by maximizing crop yields and minimizing losses due to infestations or diseases [1]. Without effective pest management, agricultural productivity would be severely compromised, leading to significant economic losses and food shortages. Approximately one-third of agricultural products rely on the application of pesticides [2]. Without their use, there would be a stunning 78% reduction in fruit production, a 54% decrease in vegetable production, and a 32% decline in cereal production [1,3].

Pesticides contribute to the sustainability of agricultural practices by allowing farmers to produce more food on less land, thereby conserving natural habitats and reducing the pressure to expand agricultural areas into ecologically sensitive areas. Additionally, pesticides help reduce crop loss by preventing damage from harmful pests and pathogens, ensuring a more abundant and reliable supply of food for consumers [4]. However, the use of pesticides also raises concerns about their potential adverse effects on human health and the environment, necessitating careful regulation and management to minimize risks while maximizing benefits. At the same time, the importance of pesticides in food production cannot be overstated, as they remain indispensable tools in the global effort to feed a growing population sustainably.

This review aims to comprehensively explore the complex issues surrounding pesticides in food production, synthesizing current knowledge and emerging trends to emphasize their impacts, regulations, and future directions. Covering the history of pesticide use, pesticide types, residues, health and environmental impacts, regulatory frameworks, emerging trends, controversies, and mitigation strategies, it provides a holistic understanding of pesticide use in food production, aiming to drive positive change.

## 2. Historical Context of Pesticide Use in Agriculture

In various forms, pesticides have been used for centuries to protect crops from pests and diseases. Ancient civilizations used natural substances like sulfur, arsenic compounds, and plant extracts to control pests and enhance crop yields [5]. The late 19th and early 20th centuries saw rapid advancements in chemistry and the development of synthetic pesticides. Compounds such as DDT, organophosphates, and synthetic pyrethroids revolutionized pest control and led to significant increases in agricultural productivity [5,6]. In the mid-20th century, we witnessed a surge in pesticide use driven by the Green Revolution and intensive farming practices [7]. However, the widespread use of pesticides raised concerns about environmental pollution, wildlife toxicity, and human health risks [8,9]. In response to growing concerns, governments worldwide began implementing regulatory frameworks to control pesticide use. Laws were enacted to assess pesticide safety, establish permissible residue levels, and regulate pesticide manufacturing, distribution, and application [10,11]. The latter part of the 20th century and the early 21st century witnessed a shift towards more sustainable pest management practices, such as integrated pest management (IPM).

Despite regulatory efforts and advances in pest management strategies, challenges persist in pesticide use. These include the development of pesticide resistance in pests [12], unintended environmental consequences [13], concerns about pesticide residues in food [14], and the need to balance agricultural productivity with environmental sustainability and human health. The relationship between technological innovation, environmental concerns, regulatory responses, and evolving agricultural practices is complex. However, continuous efforts are being made to address these challenges and promote more sustainable approaches to pest management in agriculture.

## 3. Types of Pesticides Used in Food Production

Pesticides used in food production nowadays can be classified based on various criteria, including their chemical composition, mode of action, persistence, and target pests. The most common classifications are based on target organism and chemical composition.

**Figure 1:**
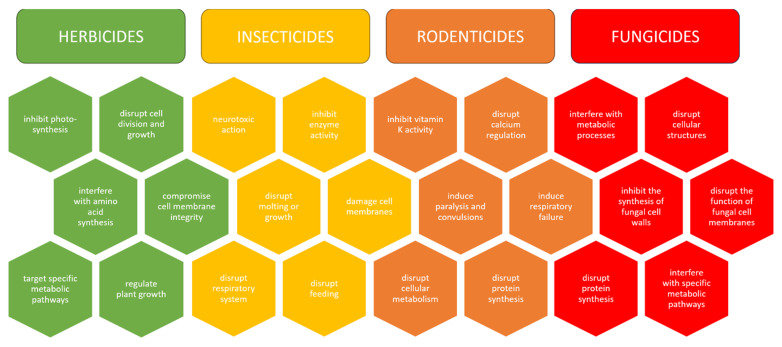
Pesticide classification based on the target organism.

Pesticides protect crops by controlling weeds (herbicides), insects (insecticides), animal infestation (rodenticides), and fungal diseases (fungicides) (Figure 1). Depending on their target and purpose, pesticides come in various formulations. 

Herbicides are the most common type of pesticide used, accounting for more than 50 percent of the pesticides used by the agriculture sector [15]. They are designed to control undesirable vegetation by inhibiting photosynthesis, disrupting cell division and growth, interfering with amino acid synthesis, compromising cell membrane integrity, targeting specific metabolic pathways, and regulating plant growth [16].

Insecticides control insect pests through neurotoxic action, inhibition of enzyme activity, disruption of molting or growth, damage to cell membranes, disruption of the respiratory system, and disruption of feeding [17].

Rodenticides are pesticides designed to control populations of rodents, primarily rats and mice, by targeting their physiology and behavior to induce mortality. The most common type, anticoagulant rodenticides, interfere with blood clotting mechanisms by inhibiting vitamin K activity, leading to internal bleeding and death. Hypercalcemia-inducing rodenticides disrupt calcium regulation, causing organ failure and death. Some rodenticides target the nervous system, inducing paralysis, convulsions, or respiratory failure, while others affect cell metabolism or protein synthesis, leading to physiological disturbances and death [18].

Fungicides are chemicals designed to control or eradicate fungal diseases in plants. They generally act directly by interfering with fungal metabolic processes or disrupting cellular structures. Some fungicides inhibit the synthesis of fungal cell walls, while others disrupt the function of cell membranes. They may also target the ribosomes or interfere with metabolic pathways essential for fungal growth and reproduction [19].

Pesticides can also be categorized based on their chemical structure and composition. Common chemical classes include organophosphates, carbamates, pyrethroids, neonicotinoids, organochlorines, triazines, and herbicides like glyphosate.

Organochlorines are synthetic pesticides that contain chlorine atoms bonded to carbon atoms in their chemical structure. Notable examples include DDT, aldrin, dieldrin, and chlordane. Organochlorines were widely used in the past but have been largely phased out due to concerns about their environmental persistence and bioaccumulation in organisms [20].

Organophosphate pesticides contain phosphorus atoms bonded to carbon atoms in their structure. They were developed as replacements for organochlorines and act by inhibiting acetylcholinesterase, an enzyme necessary for proper nerve function in insects [21]. Examples include malathion, chlorpyrifos, and diazinon.

Carbamate pesticides are similar to organophosphates but contain a carbamate functional group (-NHCOO-) instead of a phosphorus atom. They also inhibit acetylcholinesterase but typically have shorter persistence in the environment compared to organophosphates [20]. Examples include carbaryl, propoxur, and methomyl.

Pyrethroid pesticides are synthetic chemicals derived from natural pyrethrins extracted from chrysanthemum flowers. They mimic the insecticidal properties of pyrethrins but are more stable and have longer persistence [20]. Examples include permethrin, cypermethrin, and deltamethrin.

Neonicotinoid pesticides are synthetic chemicals that act on nicotinic acetylcholine receptors in insect nervous systems. They are systemic insecticides, meaning they are absorbed by plants and distributed throughout plant tissues [22]. Examples include imidacloprid, clothianidin, and thiamethoxam.

Triazine herbicides are a class of chemicals containing a triazine ring structure. They inhibit photosynthesis in plants by blocking the electron transport chain in chloroplasts [23]. Examples include atrazine, simazine, and metribuzin.

Glyphosate stands out as a separate group due to its mode of action, although it is an organophosphate. It is a broad-spectrum systemic herbicide that inhibits the enzyme 5-enolpyruvylshikimate-3-phosphate synthase (EPSPS), disrupting the synthesis of aromatic amino acids in plants. Glyphosate-based herbicides are formulated with glyphosate as the active ingredient, along with adjuvants and surfactants, to enhance the effectiveness [24].

**Figure 2:**
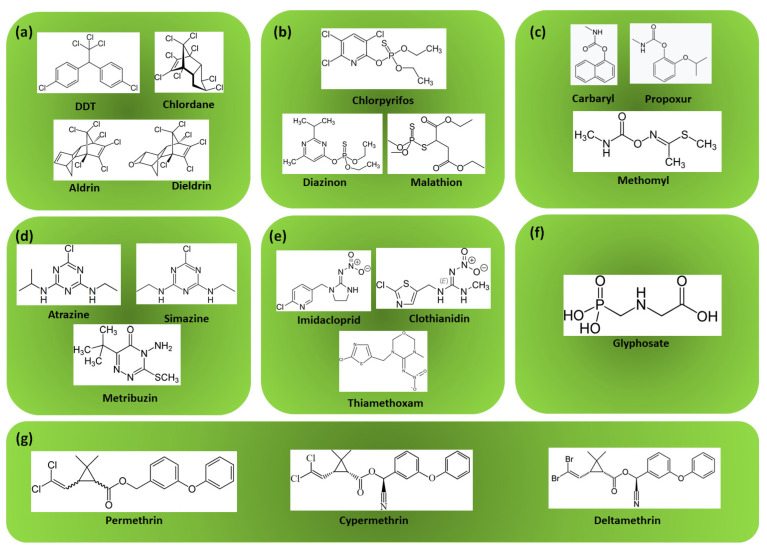
Structural formulas of the most representative pesticides from the group of (**a**) organochlorines, (**b**) organophosphates, (**c**) carbamates, (**d**) triazine, (**e**) neonicotinoids, (**f**) glyphosate, and (**g**) pyrethroids.

The structural formulas of some of the most important representatives from each pesticide group are presented in Figure 2.

## 4. Pesticide Residue in Food: Current Status

The occurrence and levels of pesticide residues in various food commodities are a significant public health concern, as pesticides are commonly used in agriculture. Pesticide residues can remain on or in the food [24], leading to potential consumer exposure. Fruits and vegetables are often the most affected, with apples, strawberries, grapes, tomatoes, and leafy greens like spinach being common examples [25]. Pesticide residues can be found on the surface as well as within the flesh of the fruit or vegetable. Animal products such as meat, milk, and eggs can contain pesticide residues if the animals are exposed to contaminated feed or treated with pesticides directly [26]. Washing and peeling can reduce pesticide residues on the surface of fruits and vegetables but may not remove residues that have penetrated the flesh [27]. Pesticides usually detected in food are from the groups of organochlorines, organophosphates, carbamates, and pyrethroids.

In agriculture, the use of pesticides is strictly regulated, with clear guidelines on which chemicals are permitted for specific crops (Table 1). However, developing countries often face challenges in implementing and enforcing strict regulations on pesticide use, resulting in higher levels of pesticide residues in food commodities [28]. These regions may lack the necessary infrastructure, resources, and regulatory frameworks to monitor and effectively control pesticide application. As a result, farmers in these areas might use higher quantities or more hazardous chemicals, leading to residues that can exceed safe levels. Additionally, the lack of education and awareness among farmers regarding the safe and appropriate use of pesticides can further exacerbate this issue, contributing to the contamination of food supplies.

This issue also leads to serious consequences for domestic markets and international trade. In 2023 and 2024 alone, several tons of fruit destined for export were returned at borders due to the presence of unauthorized pesticide residues. For example, the Netherlands rejected shipments of frozen cherries from Serbia in March 2024 due to the detection of excessive levels of dimethoate, a pesticide banned in the European Union [29]. Similarly, in August 2023, shipments of peaches and nectarines were rejected by Croatia and Montenegro due to the presence of chlorpyrifos, a pesticide that is also prohibited in the EU [30,31]. Additionally, plums from Serbia were also turned back at the border due to traces of chlorpyrifos and other banned chemicals [32]. Chlorpyrifos is still allowed in Serbia, but it is only approved for use on sugar beets under strict guidelines [33]. It is not permitted for use on other crops despite being commonly used in some parts of the world, for example, in the USA [34]. Without proper awareness and control, these restrictions do not achieve their intended purpose of protecting human health and the environment.

**Table 1:** Common pesticides used for different fruits and vegetables [35,36,37].

Crop	Insecticides	Fungicides	Herbicides
Tomatoes	Imidacloprid, Spinosad	Chlorothalonil, Copper sulfate	Pendimethalin, Glyphosate
Potatoes	Carbofuran, Imidacloprid	Mancozeb, Metalaxyl	Metribuzin, Glyphosate
Carrots	Lambda-cyhalothrin, Diazinon	Azoxystrobin, Iprodione	Linuron, Fluazifop
Lettuce	Imidacloprid, Spinosad	Boscalid, Chlorothalonil	Paraquat, Glyphosate
Broccoli	Bifenthrin, Spinosad	Azoxystrobin, Chlorothalonil	Trifluralin, Pendimethalin
Apples	Chlorpyrifos, Spinosad	Captan, Myclobutanil	Glyphosate, Paraquat
Strawberries	Malathion, Bifenthrin	Captan, Boscalid	DCPA (Dacthal), Glyphosate
Grapes	Imidacloprid, Spinosad	Mancozeb, Sulfur	Glyphosate, Simazine
Bananas	Chlorpyrifos, Diazinon	Thiabendazole, Imazalil	Paraquat, Glyphosate
Oranges	Carbaryl, Imidacloprid	Copper oxychloride, Azoxystrobin	Glyphosate, Paraquat

There is obviously growing consumer demand for food with lower or no pesticide residues, driving the market for organic and sustainably farmed products. Organic products are agricultural products grown according to strict standards that exclude or significantly limit the use of synthetic pesticides, herbicides, artificial fertilizers, antibiotics, and genetically modified organisms (GMOs) [38]. They are often misunderstood as being completely pesticide-free, but this is not the case. While organic farming avoids synthetic pesticides, it allows for the use of natural pesticides, which, like their synthetic counterparts, are designed to kill pests and can leave residues on products [39]. Because these organic pesticides break down more quickly, they often require more frequent applications, potentially leading to a higher overall pesticide load compared to conventional farming. The primary distinction between organic and conventional pesticides lies in their origin—natural versus synthetic—not in the absence of chemicals. As a result, both organic and conventional products can have pesticide residues when they reach consumers. Actually pesticide-free foodstuff, which is rarely seen, would be labeled as “pesticide-free” or “never been sprayed”, indicating that no pesticides were used at any stage of production [40]. The awareness should be raised that the assumption that organic equates to pesticide-free is a misconception, and the safety and effectiveness of these pesticides can vary just as much as those used in conventional farming.

## 5. Regulatory Framework for Pesticide Management

### 5.1. International Regulations and Standards

Over the past 35 years, international and national legal frameworks for pesticide management have progressed extensively. The Food and Agriculture Organization (FAO) adopted the International Code of Conduct on the Distribution and Use of Pesticides in 1985, later incorporating the Prior Informed Consent (PIC) procedure in 1989 and revising it in 2002. In 2013, the Code was expanded to cover public health pesticides and was renamed the International Code of Conduct on Pesticide Management, which the World Health Organization (WHO) also adopted in 2014 as its guidance framework [41]. Key international agreements, such as the Rotterdam, Stockholm, Basel Conventions, and the Montreal Protocol, have since been implemented to regulate hazardous chemicals and support the Code’s objectives. Efforts like the Strategic Approach to International Chemicals Management (SAICM) and the Globally Harmonized System (GHS) further standardized pesticide classification and labeling [41]. The Codex Alimentarius Commission (CAC), jointly established by the FAO and WHO, sets internationally harmonized food standards, which the World Trade Organization (WTO) acknowledges through its Agreement on the Application of Sanitary and Phytosanitary Measures. Among these standards, those governing maximum residue levels (MRLs) for pesticides are especially relevant. The Codex Committee on Pesticide Residues (CCPR), a subsidiary of the CAC, is responsible for developing MRLs. The CCPR sets MRLs for pesticides in foods and animal feeds, determines testing methods for MRLs, and creates a priority list of pesticides for assessment by the Joint FAO/WHO Meeting on Pesticide Residues (JMPR) [42]. In general, MRLs are set well below levels that are considered harmful to consumers. Typically, they are established with a significant safety margin, often 100 times lower than the No Observable Adverse Effect Level (NOAEL), the highest exposure level at which there are no observable adverse effects in toxicological studies. This precautionary margin is designed to account for uncertainties and variations among individuals, ensuring consumer safety even with prolonged exposure [43]. The recommended MRLs, considered safe for consumers, widely serve as food safety standards based on Good Agricultural Practices. However, despite this standardized framework, MRLs can still vary significantly across countries.

In the United States, the Environmental Protection Agency (EPA) sets MRLs in food and feed based on scientific evaluations to ensure they meet safety standards, particularly under the Federal Food, Drug, and Cosmetic Act (FFDCA). The US Food and Drug Administration (FDA) and the United States Department of Agriculture (USDA) enforce these tolerance levels, monitoring food products to ensure they comply with the established limits to protect consumer health [44]. 

In the EU, the marketing and application of plant protection products are governed by extensive legislation. A two-tier system ensures that the European Food Safety Authority (EFSA) evaluates active substances while the Member States review and approve the products at the national level. The primary regulatory framework is Regulation (EC) No 1107/2009 [45], with Regulation (EC) No 396/2005 setting legal limits on pesticide residues in food and feed [46]. EFSA is responsible for recommending MRLs of pesticides allowed in foods and feeds sold within the EU. Prior to establishing or modifying an MRL, EFSA conducts comprehensive risk assessments of pesticides to ensure food safety [47]. This assessment includes evaluating the active substance’s toxicological profile, focusing on acute and chronic health effects such as carcinogenicity, reproductive toxicity, neurotoxicity, and genotoxicity. EFSA also considers other toxicological endpoints, such as immunotoxicity, endocrine disruption, and allergenic potential. EFSA assesses exposure risks with specific attention to dietary exposure through food and water, establishing MRLs based on acceptable daily intake (ADI) and acute reference dose (ARfD) levels [47]. The risk assessment of multiple pesticide residues is a growing concern, particularly as more complex farming practices result in the use of various pesticide combinations [48]. The cumulative effects of multiple residues can be harder to predict than single pesticide exposure. In the last decade, EFSA has developed models to assess the combined risks from exposure to multiple pesticides in food, considering factors like toxicity and dietary intake patterns [49,50]. Cumulative risk assessment (CRA) is a critical approach in the pesticide evaluation process, which assesses the combined health risks from exposure to multiple pesticides with similar modes of toxicological action. This method accounts for the possibility that multiple pesticides, each at low or individually safe exposure levels, may collectively pose a significant health risk due to their cumulative effects. EFSA groups pesticides into Cumulative Assessment Groups (CAGs), where each group is based on a shared toxicological endpoint, such as neurotoxicity or endocrine disruption. For each CAG, EFSA assesses combined exposure levels from various sources, including food and water, and compares these levels to safety thresholds using the Margin of Exposure (MOE) [51]. This comparison determines if cumulative exposure remains within safe limits.

The assessment of the effects of combined exposure to multiple pesticide residues presents a significant challenge due to their potential interactions [48]. There is limited scientific data regarding complex patterns of combined exposure to multiple residues, which can result in their additive, antagonistic, or synergistic effects affecting human health. Hence, there is an urgent need to establish methods to assess these effects prior to confirming the overall risk. Concerning mixture risk assessment (MRA), Regulation (EC) No 396/2005 mandates that the dietary risk assessment of pesticide exposure should consider cumulative and synergistic effects in determining pesticide MRLs when suitable methods become available [46]. Advances in pesticide residue detection and degradation methodologies are crucial for predicting pesticide behavior in different environments, helping to improve regulatory compliance and reduce risks to human health [52]. Risk assessment continues to be a priority, with new methods being developed to predict pesticide residue degradation and its impact on human health [53]. In 2023 and 2024, updates to methodologies and tools were implemented to enhance transparency and improve CRA. EFSA refined the use of the Monte Carlo Risk Assessment (MCRA) platform, which supports evaluating the combined health impacts of multiple chemicals over time [54].

In 2020, the European Commission introduced a plan under the European Green Deal and its Farm to Fork Strategy, aiming for a 50% reduction in the use of hazardous pesticides by 2030 and at least 25% of the EU’s agricultural land should be under organic farming [55].

The strategy encourages the adoption of sustainable farming techniques, including organic farming and IPM. The IPM is a strategy introduced by the EU in 2009 under Directive 2009/128/EC, also known as the Sustainable Use of Pesticides Directive (SUD) [56]. This strategy emphasizes pest control through sustainable biological, physical, and other non-chemical methods, aiming to reduce health and environmental risks linked to conventional pesticide use. The proposal to convert the SUD (2009/128/EC) into a directly enforceable regulation, ensuring uniform application across all EU Member States, was withdrawn in March 2024. As a result, the existing Directive (2009/128/EC) remains in force, continuing to promote pesticide reduction measures but without enforceable mandatory targets [57].

One of the primary legal acts governing organic farming in the EU is Regulation (EU) 2018/848 [58], which sets the standards for organic production, labeling, and certification within the EU. It aims to ensure the sustainability of organic farming, promote high environmental and animal welfare standards, and ensure the integrity of organic products across EU member states [58]. Biopesticides (derived from natural sources like plants, fungi, bacteria, or minerals) play a significant role in organic farming, contributing to pest management in a way that supports environmental health and sustainable agricultural practices. Biopesticides in the EU are regulated under Regulation (EC) No. 1107/2009, which governs the approval of active substances in plant protection products [59]. This regulation applies to all pesticides, including biopesticides, and requires comprehensive safety assessments. The EFSA conducts risk assessments for biopesticides, focusing on their potential impacts on human health, non-target organisms, and the environment.

The regulation of pesticides and biopesticides in organic farming faces unique challenges, primarily because organic agriculture is committed to minimizing synthetic pesticides while addressing crop pests and diseases. The biopesticide registration process can be lengthy and costly, requiring data on efficacy, toxicity, environmental impact, and residue levels, similar to synthetic pesticides. In addition, biopesticides often work more slowly or less reliably than synthetic ones, which can be a challenge for organic farmers who need timely and effective solutions [60].

The transition to sustainable agricultural practices requires addressing both current pesticide use and the lingering effects of past applications. The achievement of the targets set by the EU may be impeded by the persistence of legacy pesticides from historical applications, which can remain in the environment for decades [61]. Current EU policy does not fully address the challenges posed by legacy pesticides or the need for their remediation. Identifying the pesticide mixtures in the soil is essential for conducting thorough pesticide risk assessments and evaluating their impacts on soil health [62] and, consequently, on food safety and human health. Current risk management strategies should include an assessment of DDT, chlordane, and other organochlorine pesticides, which, although banned worldwide, persist in soils and sediments due to their long half-lives, posing risks to human and environmental health [63]. The detection of banned pesticides in water samples, even after 25 years, underscores the need for improved EU policies targeting legacy pesticide remediation to meet future environmental and public health goals [61].

### 5.2. National Regulatory Approaches

The structure and content of pesticide legislation in each country are influenced by its legal system, constitution, international obligations, existing laws, institutional capabilities, and government priorities and resources. Additionally, the legislation reflects the country’s economic and social conditions, including factors like the types of crops cultivated, pest issues, vector-borne diseases, dietary habits, type of pesticides needed, environmental conditions, etc. By carefully considering these elements, countries can create an effective legal framework for pesticide management that aligns with national needs [64].

In 2020, FAO and WHO provided guidance on pesticide legislation intended to help countries develop effective pesticide legislation and institutional structures [41]. The Code of Conduct advises governments on developing national pesticide management legislation, focusing on comprehensive regulation and effective enforcement. It identifies key areas for regulation, encourages programs like IPM, and aligns with Codex Alimentarius standards, which set internationally recognized MRLs in food endorsed by the WTO. Codex MRLs, often legally adopted by governments, ensure national alignment with international safety standards. National approaches to MRLs for pesticides vary widely across countries and are influenced by factors such as public health policies, agricultural practices, regulatory frameworks, and international trade considerations. In collaboration with the WHO, the FAO does not interfere directly in national MRL settings but influences them by providing scientifically grounded, internationally recognized standards through Codex, which countries can adopt or use as a reference for their own MRL policies. Many countries align their MRLs with Codex standards, especially for traded goods, but they retain the autonomy to set stricter or more lenient MRLs based on national policy priorities [65].

Initially, many developing countries lacked pesticide regulations, with limited awareness of pesticide hazards and increased use of highly toxic products. Progress has been made, but existing laws often lack full alignment with international standards, have outdated penalties, and face enforcement challenges, particularly for resource-limited governments [41]. A critical concern in the field of food safety is the absence of globally harmonized pesticide regulations and safety criteria [66]. Discrepancies in regulatory standards among developed and developing countries also create significant trade barriers, as numerous developing countries employ some of the non-approved pesticides or maintain different MRLs. While most developed countries have implemented their own MRL frameworks, compliance with these standards presents substantial challenges for developing countries [67].

Governments are encouraged to develop policies and legislation regulating pesticides across their life cycles, aligning with FAO, WHO, and binding international standards. In meeting international obligations, countries may incorporate treaties into national laws by reference, directly transpose their text, or adjust legislation to reflect the treaty’s goals in a manner suited to national policies and resources. Effective implementation often requires tailoring based on local practices, as with agreements like the Rotterdam and Basel Conventions, which introduce specific obligations for export and health-impact reporting [41].

In general, a comprehensive pesticide law should include a clear scope and definitions that align with international standards. Key provisions should mandate a registration system for all pesticides, stipulate application procedures, establish licensing and inspection protocols, and regulate packaging, labeling, and responsible pesticide use. The law should also define import/export, transport, storage, and disposal standards, emphasizing alignment with international safety standards. It must designate authorities for monitoring, inspecting, and reporting pesticide incidents and include penalties and procedures for offenses, ensuring legal coherence within national frameworks [41].

## 6. Health and Environmental Impacts of Pesticide Exposure

### 6.1. Human Health Effects

Exposure to pesticides, whether through direct contact, residues in food, or environmental contamination, can lead to acute and chronic health effects, underscoring the importance of sustainable and responsible pesticide use.

Agricultural pesticide exposure presents a significant public health risk, particularly in developing countries. The WHO estimates that annually, around 3 million workers in developing countries suffer from severe pesticide poisoning, leading to approximately 18,000 deaths [68]. Because pesticides’ mechanisms of action often affect multiple species, they pose significant risks to human health due to potential carcinogenic, cytotoxic, and mutagenic properties. [69].

The toxicity of a pesticide formulation is determined by its active ingredients and may also be influenced by synergistic or inert compounds that enhance or modify its toxicity. While the exact molecular mechanisms behind how pesticides induce biochemical changes are not completely understood, studies suggest that most pesticides induce oxidative stress [69]. In general, pesticide exposure generates reactive oxygen species (ROS) and reactive nitrogen species (RNS), which can activate at least five distinct signaling pathways, including mitochondria-mediated apoptosis. Additionally, these pathways weaken antioxidant defenses and cause damage to proteins, lipids, and nucleic acids, disrupting cellular signaling and contributing to long-term health risks [70].

Acute and chronic exposure to pesticides represents a significant health concern worldwide, with consequences ranging from immediate toxic effects to long-term health complications. Symptoms of acute pesticide poisoning can manifest within minutes to hours following exposure and can vary depending on the pesticide’s toxicity and exposure route (inhalation, ingestion, dermal, or ocular). Symptoms may include gastrointestinal symptoms (nausea, vomiting, diarrhea, abdominal cramps), neurological symptoms (headaches, dizziness, confusion, seizures, coma), cardiovascular symptoms (bradycardia, hypotension, cardiac arrest), respiratory symptoms (breathing difficulty, excessive bronchial secretions, and respiratory failure) and others, such as urinary incontinence, excessive salivation, etc. [71]. These effects can emerge rapidly, and without timely and proper treatment, they may result in severe complications or death. Such acute effects are widespread among agricultural workers, especially in regions where pesticide application is frequent and safety measures are inadequate.

On the other hand, chronic exposure, whether occupational, environmental, or through indirect dietary sources, involves prolonged or repeated contact with pesticides at lower levels over time. This type of exposure is associated with more harmful health risks, which may manifest after years of cumulative exposure. In the general population, a diet represents the primary route for pesticide exposure, predominantly through residues present in fruits, vegetables, and animal-derived food products [72]. In addition, pesticides may contaminate drinking water. Several studies from the Netherlands, Denmark, and Ireland reported the presence of pesticides in drinking water at concentrations exceeding quality standards [73].

Chronic pesticide exposure has been linked to a range of severe conditions, including cancer (leukemia, multiple myeloma, and cancers such as breast, bladder, colon, liver, lung, and brain cancer), neurological disorders (Parkinson’s and Alzheimer’s disease), respiratory disorders (asthma, particularly in children), diabetes, and endocrine disruption, which can lead to reproductive issues and developmental defects [69]. It has been reported that organochlorines and organophosphates disrupt the nervous system and are associated with severe health conditions, including cancer and various organ damage [74]. Carbamates, which are generally considered less toxic, are associated with respiratory problems and developmental disorders, while pyrethrins and pyrethroids can negatively affect neurocognitive development in children and are linked with an increased risk of diabetes and leukemia [69,74].

The overall burden of disease caused by pesticide exposure in Europe, either for the general population or specific groups, is currently impossible to estimate. However, strong or suspected associations have been identified between pesticide exposure and an increased risk of several chronic health conditions [75]. Although identifying the exact active substances responsible is challenging, certain pesticide groups, such as legacy pesticides (organochlorines, e.g., DDT and lindane), as well as organophosphates and pyrethroids, have been associated with a heightened risk of various chronic diseases.

A human biomonitoring study (HBM4EU) conducted from 2014 to 2021 revealed that 84% of samples taken from children and adults across five European countries contained residues of two or more pesticides. The study identified at least 46 pesticides and their metabolites. Over 90% of samples showed traces of pyrethroids and the banned chlorpyrifos. Glyphosate and its metabolite aminomethylphosphonic acid also showed widespread, though low, concentrations across the EU [72]. Monitored pesticides were detected in higher concentrations in children than adults [76], which altogether emphasized a need for evaluating the effectiveness of policies aimed at reducing pesticide use and associated risks.

Recent data from the EFSA indicate that dietary exposure to individual pesticides is unlikely to pose a risk to consumer health. The 2021 EFSA annual report on pesticide residues in food revealed that 96.1% of samples were below the MRL, while 3.9% exceeded it, with 2.5% being non-compliant [77]. These rates improved compared to 2020. However, samples from non-EU countries exhibited significantly higher rates of MRL exceedance (10.3%) and non-compliance (6.4%) than those from the EU (2.1% and 1.3%, respectively). In 2022, 96.3% of the samples analyzed were below the MRL, while 3.7% exceeded it; 2.2% of these were classified as non-compliant after accounting for measurement uncertainty [78].

In general, dietary exposure assessments for acute and chronic risks showed a low overall health risk to consumers. However, as stated, EFSA faces challenges in assessing dietary pesticide risks, including evaluating cumulative effects of multiple residues, variability in residue levels, limited data on long-term exposure, and differences in dietary habits. EFSA aims to improve regulatory control systems to maintain high consumer protection standards, complete cumulative risk assessments for all relevant pesticide residues by 2030, and harmonize risk assessment practices across EU member states through updated guidance and workshops [54].

Globally, the impact of pesticides on health is a significant concern, particularly in developing countries with less stringent regulations and enforcement than the EU. International efforts, such as those by the WHO and FAO, aim to promote safer pesticide use, improve monitoring, and reduce reliance on harmful pesticides through sustainable agricultural practices. However, significant challenges remain in ensuring health protection globally [66].

### 6.2. Ecological Consequences and Biodiversity Concerns

The widespread use of pesticides in modern agriculture has significant environmental impacts, particularly concerning the health of ecosystems and biodiversity. While essential for controlling pests and enhancing crop yields, pesticides often leave residues on plants that can settle into the soil, run off into waterways, or leach into groundwater [74]. This contamination poses a substantial threat to both terrestrial and aquatic ecosystems. For example, a 2014 study found glyphosate in most rivers, streams, and wastewater treatment plants across 38 U.S. states, as well as in 70% of rainfall samples [79,80]. Even when present at levels considered safe, pesticides can disrupt ecosystems by reducing biodiversity, including the decline of beneficial insects, birds, and amphibians.

One of the most significant ecological consequences of pesticide exposure is biodiversity loss. Pesticides can kill non-target species, including important pollinators such as bees, butterflies, and other insects that play a critical role in maintaining healthy ecosystems [81]. Neonicotinoids, a popular class of insecticides that target the nervous system of insects, have been linked to the decline of wild and domesticated pollinators. Studies have shown that bumblebee colonies exposed to neonicotinoids grow more slowly and produce fewer queens, while honey bees suffer from impaired learning, communication, and colony growth [82,83]. The decline of pollinators is particularly concerning, as they are essential for the production of many fruit, nut, and vegetable crops.

In addition to harming pollinators, pesticide exposure can have cascading effects on entire ecosystems. For instance, the overuse of herbicides has led to the emergence of “superweeds”—weed species that have developed resistance to multiple herbicides, making them difficult to control [84]. These superweeds can overrun agricultural fields, outcompeting crops and reducing yields. The increasing prevalence of herbicide-resistant weeds has forced farmers to rely on older, more hazardous chemicals, further exacerbating environmental contamination.

The ecological impact of pesticide resistance is not limited to plants; it also affects insects and other organisms. Overuse of insecticides can cause non-target species to develop resistance, increasing their populations and disrupting the balance of ecosystems. For example, the widespread use of agricultural insecticides has contributed to the spread of insecticide-resistant mosquitoes, complicating efforts to control diseases such as malaria [85]. This mirrors the public health crisis of antibiotic resistance, where the overuse of antibiotics has led to the emergence of drug-resistant infections.

Considering all this, it is clear that the environmental impacts of pesticide exposure are far-reaching, affecting not only agricultural systems but also the broader ecological balance. The loss of biodiversity, the emergence of pesticide-resistant species, and the contamination of natural resources are serious concerns that highlight the need for more sustainable pest management practices in agriculture.

## 7. Strategies for Mitigating Pesticide Risks

### 7.1. Integrated Pest Management (IPM) Strategies

The development of IPM was induced by the widespread negative consequences of excessive pesticide use, including environmental degradation, the development of pest resistance, and concerns about human health. The primary goals of IPM are multifaceted, with economic and environmental objectives. Unlike traditional pest control methods that aim for complete eradication, IPM focuses on maintaining pest populations below economically damaging levels, thereby reducing the cost of management for farmers and minimizing the ecological impact of pesticides. The IPM aims to create a more sustainable, effective, and environmentally friendly approach to pest control by reducing reliance on chemical pesticides, promoting the use of alternative methods, and ensuring that pest management practices are economically viable and socially acceptable. Thus, IPM is not solely focused on economic gains but balances economic, health, and environmental priorities.

The concept of integrated control emerged in 1959 [86] as a response to the challenges associated with heavy reliance on chemical control measures. IPM’s principal component is the use of multiple control tactics to manage pests effectively while minimizing pesticide use and associated costs. IPM does not exclude the use of pesticides but rather integrates alternative control methods with periodic monitoring to reduce pesticide application to necessary levels only. This strategy can be applied to a single pest or a group of pests within an ecosystem or across an entire agricultural community, with varying levels of integration depending on the production scale.

Two key concepts in IPM are the Economic Injury Level (EIL) and the Economic Threshold (ET). The EIL is the lowest pest density that causes economic damage, where the management cost equals the profit loss from damage. The ET, set below the EIL, is the point at which management actions are taken to prevent pest densities from reaching the EIL. These thresholds are determined through research on the relationship between pest densities, crop damage, yield losses, and management costs [86,87].

The levels of IPM integration are categorized into four stages: Level 1 focuses on managing individual pest species or species complexes; Level 2 involves managing a community of pest species, including insects, pathogens, and weeds; Level 3 addresses ecosystem-level management by incorporating both crop and non-crop host plants and other components; and Level 4 extends to community-wide management, considering social and economic factors within the farming community [88,89]. These levels provide flexibility for producers to tailor IPM practices to their specific needs and capacity, making IPM adaptable to various production systems by considering a wide range of biotic and abiotic factors.

IPM incorporates several types of control measures, including cultural, biological, mechanical, chemical, and behavioral controls. Cultural control involves farm management strategies and the use of resistant plant varieties to minimize pest impact, such as crop rotation to disrupt pest life cycles and sanitation practices to reduce pest pressure. Biological control focuses on protecting or introducing beneficial species, like reducing broad-spectrum pesticide use to preserve predators or introducing natural enemies like ladybugs in greenhouses, though it is important to consider the potential ecological risks associated with the introduction of exotic species. Mechanical control employs physical measures to trap, exclude, or eliminate pests, such as using grease bands on fruit trees or trap crops to protect fields. Chemical control, used as a last resort, targets pests specifically, often utilizing periodic sampling and action thresholds to minimize applications. Behavioral control alters pest behavior through pheromones or semiochemicals to disrupt activities like mating [86].

IPM has proven to be effective in many cases. The benefits include the potential for reduced management costs, decreased pesticide use, and the promotion of more sustainable practices by minimizing reliance on chemical control. By employing multiple control tactics, IPM reduces selection pressure from pesticide dependence, thereby slowing or preventing the development of pesticide resistance. High IPM adoption areas have documented reductions in pesticide residues on products and in surface waters, attributed to overall decreased pesticide use and the adoption of newer, less persistent pesticides. IPM’s targeted chemical management narrows the spectrum of activity to specific pests, helping to increase populations of natural enemies, which can keep pest numbers below action thresholds and reduce the need for chemical applications. In many cases, IPM adoption has been associated with increased incomes and yields, where net profits were significantly higher than in conventional production while also reducing pesticide use [90,91,92]. However, there are challenges; in some instances, pesticide usage or costs have increased due to more frequent field monitoring or the use of newer, more expensive pesticides [90]. The effectiveness of IPM varies by region and crop, and while it can be implemented universally, the extent and success depend on local conditions and the availability of research. Continued research and dissemination are crucial to improving IPM adoption, as well as the level of commitment to and understanding of IPM practices by those implementing them.

### 7.2. Alternative Approaches to Conventional Pesticide Use and Their Role in Modern Agriculture

As the environmental and health concerns related to conventional pesticide use grow, alternative methods are being explored to minimize the negative impacts on ecosystems, human health, and biodiversity. These approaches offer more sustainable, environmentally friendly, and often cost-effective solutions for pest management in agriculture. However, their effectiveness, costs, and practicality vary, and many challenges remain in entirely replacing conventional chemical pesticides.

One of the most widely used techniques is biological control, which involves leveraging natural predators, parasites, or pathogens to regulate pest populations. This approach avoids the use of harmful chemicals while helping to preserve ecological balance. For example, predatory insects such as ladybugs and lacewings feed on aphids and other pests, while parasitic wasps lay eggs inside pests like caterpillars, effectively controlling their growth. Additionally, microbial insecticides, such as *Bacillus thuringiensis* (Bt), target specific pests without affecting beneficial organisms or non-target species. These methods have proven effective in the sustainable management of pest populations [93].

Botanical and natural pesticides offer another promising solution for reducing chemical inputs in agriculture. Derived from plants, minerals, or other natural sources, these pesticides break down more quickly in the environment and pose less risk to human health and ecosystems. Examples include neem oil, which disrupts the life cycles of a wide range of pests [94], and pyrethrin, extracted from chrysanthemum flowers, which serves as a contact insecticide [95]. Diatomaceous earth, a mineral-based powder, can be used to physically damage pests’ exoskeletons, leading to their death [96]. These natural alternatives provide effective pest control with a lower environmental footprint.

Pheromone traps and mating disruption represent innovative pest control methods that target specific pest behaviors. Pheromones, which insects use for communication, can be synthesized to lure pests into traps or confuse them during mating [97]. Farmers can prevent male insects, especially moths, from finding mates by dispersing synthetic pheromones in crop fields, effectively controlling population growth. This method disrupts the reproductive cycle of pests without harming non-target species, making it an eco-friendly approach.

Genetic engineering and pest-resistant crop varieties are also promising in reducing the need for chemical pesticides. Genetically modified (GM) crops like Bt cotton and Bt corn are designed to produce proteins from *Bacillus thuringiensis* that are toxic to specific pests but safe for humans and other animals [98]. In addition, conventional breeding and gene editing techniques have enabled the development of pest-resistant crop varieties. These crops are inherently less attractive to pests, reducing the need for frequent pesticide applications and lowering environmental contamination.

Another pillar of sustainable pest management is the use of cultural practices and agroecological techniques. These farming methods focus on naturally reducing pest pressures by promoting ecosystem health and biodiversity. For example, crop rotation prevents the establishment of pest populations, while cover cropping improves soil health and reduces weed competition. Polyculture, or the practice of planting diverse crops together, decreases the likelihood of attracting pests, while mulching and solarization help suppress weeds and soil-borne pests. Agroecology encourages resilient ecosystems that are less dependent on chemical inputs [99].

Lastly, physical and mechanical controls are effective alternatives to chemical pesticides. These methods include using physical barriers like row covers and nets to prevent pest access to crops, as well as mechanical techniques such as tillage or vacuuming pests from plants. By physically excluding or removing pests, these approaches provide a direct and non-chemical way to protect crops [100].

Postharvest grain storage could play a significant role in minimizing pesticide residues and ensuring food safety. Alternative strategies such as grain bulk aeration, low-temperature storage, and controlled atmosphere storage should be fully adopted to reduce the reliance on chemical treatments. These methods preserve grain quality and could significantly contribute to safer and more sustainable storage practices, aligning with the growing demand for residue-free food products.

In the past decade, green technologies such as ozone treatment and cold plasma have gained significant attention for their potential in reducing pesticide residues in food. As a strong oxidizing agent, ozone can degrade pesticide residues on food surfaces without leaving harmful by-products [101]. Similarly, cold plasma technology has emerged as an effective, non-thermal method for degrading pesticide residues while maintaining food quality [102].

Precision agriculture technologies, particularly smart herbicide application, have emerged as an effective strategy for reducing pesticide use while maintaining crop protection [103,104]. Smart sprayers equipped with advanced sensors and artificial intelligence can precisely target weed plants, minimizing herbicide application to non-target areas. This approach significantly reduces the overall pesticide burden on agricultural fields, leading to lower environmental contamination, reduced impact on non-target organisms, and improved soil and water quality.

Nowadays, nanopesticides offer exciting possibilities, enhancing the delivery, stability, and effectiveness of pesticides by employing nanotechnology [105]. These nanoformulations provide a controlled release and improved targeting, potentially reducing the quantity of pesticide required. Yet, they are still in development, and widespread field use remains limited. While these alternatives help mitigate the environmental damage caused by synthetic pesticides, their slower action, complexity, and diverse effectiveness make them less suitable for severe infestations or large-scale farming compared to conventional pesticides.

Cost is a significant factor when comparing alternative strategies to conventional pesticides. Biological control, for example, requires higher initial setup costs, such as the introduction and maintenance of beneficial organisms. IPM demands more labor and training for pest monitoring, often requiring significant investment. Nanopesticides, though promising, currently involve high production costs due to their advanced manufacturing processes. While their long-term use may lower costs by reducing pesticide volumes and improving efficiency, they remain more expensive than conventional formulations at present. Similarly, natural pesticides, though cheaper in some cases, tend to degrade faster, increasing the need for repeated applications, which raises labor and overall product costs.

Despite the growing availability of alternative pest management strategies, conventional pesticides continue to dominate modern agriculture. Their primary advantage is their immediate effectiveness. Chemical pesticides offer fast-acting, broad-spectrum control, which is crucial in severe infestations where timely intervention is necessary to prevent significant crop losses. Moreover, conventional pesticides are supported by an established infrastructure and supply chain, making them readily available and easy to apply. Farmers are familiar with their use, and less specialized knowledge is required compared to implementing complex systems like IPM. In addition, the low cost of conventional pesticides compared to alternatives makes them the default choice for many agricultural processes. Another reason for the persistence of conventional pesticides is the lack of alternatives for specific pests. Some pests are highly resilient, and the use of conventional pesticides is often the only reliable option. Over time, the development of pest resistance to chemical pesticides drives innovation, but even in resistance management, chemical solutions are frequently reformulated to maintain efficacy. From all mentioned, it is obvious that reliance on conventional pesticides makes it unlikely that alternative methods will be widely embraced by farmers in the near future.

But what can be done to address this critical issue? One apparent solution to the current pesticide crisis has been largely overlooked. It is logical and essential, especially since eliminating pesticides is still impossible. The use of pesticides must be strictly regulated, and their access should be restricted to trained and certified professionals. Like many other technological advancements, pesticides can significantly benefit our lives but become hazardous when misused. Therefore, a comprehensive approach that combines regulation, education, and enforcement is crucial to minimize risks while maximizing benefits. To illustrate this point, parallels can be drawn from other technologies like cars, mains electricity, and medicines [4]. Cars provide independent transportation for people but also have negative aspects such as accidents, emissions contributing to greenhouse gases, and inefficiency compared to public transportation alternatives like buses or trains [106]. However, despite these drawbacks, many people still choose to use cars due to their convenience. To mitigate the risks associated with cars, regulations are implemented that make them safer and less polluting, and drivers must pass proficiency tests. Similarly, mains electricity is known for its undeniable benefits but also its negative impacts, including pollution during production and the risk of accidental electrocution. Despite these risks, the benefits of electricity are deemed irreplaceable. The most obvious examples are medicines, which have a key role in reducing disease and preserving life but also pose hazards if not used carefully [4,107]. These examples draw parallels with pesticides, as they represent technologies that improve our lives, provided they are subject to regulation and utilized in a manner where the benefits substantially outweigh the risks. The need for responsible regulation and usage is urged to optimize the positive impacts while mitigating the negative consequences associated with these technologies. Only individuals who have undergone proper training and education should be authorized to use pesticides. It would ensure that those handling pesticides understand the potential risks and are equipped to apply them in ways that minimize harm to the environment and human health. Such regulations would reduce improper use, accidental exposure, and environmental contamination, making pesticide use more responsible and controlled. By limiting access to trained individuals, we can create a safer, more sustainable approach to pest management while still allowing the necessary use of these chemicals where alternative methods may fall short.

## 8. Controversies Surrounding Pesticide Use in Food Production—Public Perception and Consumer Concerns

The use of pesticides has long been surrounded by various controversies, raising significant concerns across environmental, health, and regulatory domains. One of the primary issues is their environmental impact. Pesticides often cause unintended harm to non-target species, contaminate soil and water, and disrupt ecosystems [108]. Runoff from pesticide-treated fields can pollute nearby water sources, damaging aquatic life and biodiversity. Moreover, human health risks are a major concern, particularly for agricultural workers and those living near areas where pesticides are heavily used. Prolonged exposure to certain pesticides has been linked to serious health problems, including cancer, neurological disorders, respiratory issues, and reproductive harm [109]. Another growing issue is pesticide resistance. The overuse of pesticides can lead to pests developing resistance, making the chemicals less effective over time [110]. This creates a cycle where increasingly higher doses or stronger pesticides are required, further escalating the chemical burden on the environment. Additionally, pesticides can negatively affect beneficial organisms like pollinators, natural pest predators, and essential soil microorganisms, disrupting ecological balances and even leading to secondary pest outbreaks [108,111].

There are also significant regulatory and ethical concerns. The disposal of used pesticides and their containers is a critical environmental concern, particularly in undeveloped countries where improper disposal methods are common. Many question whether current pesticide regulations adequately protect human health and the environment. One of the most important ethical issues is the export of banned pesticides. Countries like those in the EU have banned certain harmful pesticides domestically but continue to produce them for export to nations with looser regulations [112,113,114]. This practice has been criticized as undermining international efforts to promote safer pest management and raises questions about the global responsibilities of pesticide-producing countries [115]. This global trade of hazardous chemicals, driven by economic and trade agreements, further complicates efforts to standardize safety measures and reduce environmental and health risks worldwide.

Some regulatory agencies have been criticized for being influenced by industry interests, potentially compromising public safety in favor of profit [116]. This also ties into the controversy over the speed at which new pesticides are approved. Critics argue that in the race for profit, some chemicals may be introduced too quickly without sufficient long-term testing on their environmental and health impacts. As older, more toxic pesticides are phased out, newer chemicals are being introduced. These may have different environmental and health impacts, which are still being studied. One of the latest such examples is glyphosate, one of the most widely used herbicides globally, which was first introduced to the market in 1974. The controversy surrounding glyphosate stems primarily from concerns about its potentially harmful effects on human health and the environment [117,118]. A major argument is whether glyphosate was adequately tested before its widespread use. Some critics argue that it was introduced too quickly, without sufficient long-term studies on its safety. This concern gained traction after the World Health Organization’s International Agency for Research on Cancer (IARC) classified glyphosate as “probably carcinogenic to humans” in 2015 [119]. This classification sparked widespread debate and led to numerous lawsuits, with plaintiffs claiming that glyphosate exposure caused cancer, particularly non-Hodgkin’s lymphoma. On the other hand, regulatory agencies such as EPA and EFSA have stated that glyphosate is unlikely to pose a carcinogenic risk to humans when used according to guidelines [120,121]. However, the conflicting assessments have fueled public mistrust and ongoing legal battles, making glyphosate a highly controversial topic in the fields of agriculture, public health, and environmental policy.

## 9. Conclusions

While alternative strategies for pest management are being developed and somewhat hold promise, conventional pesticides remain essential to meeting the global food demands of a rapidly growing population. Despite the ongoing efforts to innovate and reduce chemical inputs, completely replacing synthetic pesticides is not yet feasible. Therefore, it is crucial to implement stricter regulations to govern pesticide use. Those authorized to handle pesticides should undergo proper training to ensure they are applied safely and responsibly, reducing risks to human health and the environment. Additionally, new pesticides should not be rushed to market. Rigorous, long-term studies should precede any widespread adoption to fully understand their potential impacts. While these measures may not entirely resolve the challenges posed by pesticides, they represent a pragmatic approach to balancing agricultural productivity with environmental and public health concerns.

## Data Availability

No new data were created or analyzed in this study.
